# McGill Society for Study of Comparative Psychology

**Published:** 1896-06

**Authors:** 


### PROCEEDINGS FOR THE YEAR 1894–95 OF THE SOCIETY FOR THE STUDY OF COMPARATIVE PSYCHOLOGY IN CONNECTION WITH THE FACULTY OF COMPARATIVE MEDICINE AND VETERINARY SCIENCE OF McGILL UNIVERSITY, MONTREAL.

(Concluded from page 418.)

During the discussion which followed the President compared the mental development of whites and negroes in one of the Bahama islands which he visited several years ago. The children attended the same schools, and thus under similar environment it was found that up to a certain age the negro children proved the more apt scholars, but that age being passed, they soon fell behind their fairer schoolmates. This precocity of mental development followed by early arrest has been observed and commented upon by those who have undertaken the education of the negro—there is great aptitude in the beginning, but inability to go on to original thinking. In reply to a question from Mr. MacNider, he also stated that, in some respects at least, the negro matured physically at an earlier age than the Caucasian. Mr. Zink, in referring to the subject of the paper, said that the relation between æstheticism and religion was greater than might at first appear, and was a matter worthy of thought and investigation.

The President then read a report of the recent meeting of the American Psychological Society, held at Princeton, at which he was present and read a paper on "The Psychic Development of Young Animals and Its Physical Correlation."

This meeting was well attended, the papers presented covering a wide range of psychological topics. Many of the subjects were treated from the comparative point of view, which is, indeed, the true method. Prof. Jastrow has said, "in proportion to our knowledge of the earlier, simpler, and lowlier forms of intelligence will be our ability to appreciate and utilize the best and worthiest faculties in ourselves."

After the reading of the minutes of the previous meeting, "Our Duty to the Lower Animals," the subject of a paper by Mr. S. L. Cleaves, was treated in a thoroughly original manner. The claim of animals to the kind consideration of man, who frequently misjudged their actions, was referred to at length. The cruelties to which horses are subjected in conformity to the dictates of fashion, such as docking, the use of tight check-reins, and powerful curb-bits, are matters about which owners are in many cases very careless, and many of the abuses inflicted on cab horses could be prevented by the public, who are singularly thoughtless in this respect. Referring to the increasing humane treatment of animals, the writer expressed the hope that the next few years would witness a still greater advance. The veterinarian, in the practice of his profession, can do much in the way of awakening and fostering a sentiment of kindness to the domestic animals. In conclusion he referred to the growth of this Society under the the able direction of the President, and congratulated the members on the work it has done since its inauguration.

Mr. J. C. Cutting read a paper on "Expression in Animals," describing at length the various methods made use of in the communication of thought. These modes of expression have been developed largely as a result of the relations existing between the lower animals and man. The expression by countenance is not so marked as in man, yet there are many indications by which we can discern the condition of the mind not only in the normal, but also in the abnormal states. It has been stated that different facial expressions in man are effected by impulses along different fibres of the facial nerve. Spencer says: "When any quantity of nerve force is produced and we have a state of feeling, it must expend itself somewhere. As when the cerebro-spinal system is excited, the nerve force expends itself in sensations, active thought, or movements, and, if undirected, will take the most habitual route, and the facial muscles come into play." Gestures are closely related to attitude and posture, each of which is indicative of the mental state of the animal. This is especially noticed in birds and carnivora, in which we see erection of the dermal appendages when angry or frightened. The mobile ears of the horse are organs of expression, a fact apparent to anyone who takes but even a passing interest in this animal. Articulate sounds as a means of communication constitute a later form of expression, from an evolutionary point of view, the other forms being more primitive in nature.

Mr. H. D. Clarke followed with a paper on "Bovine Intelligence." First comparing the brain of the ox with that of a man, he proceeded to show the relation existing between the intelligence of the animal and its environment. The most intelligent cattle are found among the smaller breeds, the Jerseys and Devons. An instance was related of a cow, which appeared to have an idea of the time of day and also understood a command. On one occasion when going out through the wrong door she was told "The other door, Molly," and turning immediately went out by the right one. It was believed that, while she did not comprehend the meaning of the words, she interpreted them as a correction for what she was about to do, and turned to the other door. The instinctive feigning of calves was referred to and the development of intelligence described. The paper was concluded by the statement that cattle are more intelligent than is generally supposed by those who are not brought into close relations with them.

During the discussions which followed the reading of these papers the question as to whether fox-hunting is a cruel pastime or not, and whether it should be followed by the true sportsman, was dealt with. No conclusions were formulated by the members, and the President advised a reconsideration of the question at some future meeting.

After the reading of the minutes of the previous meeting, Mr. B. K. Baldwin presented a paper on "The Relations Between the Intellectual Status of the Owner and the Horse." The writer endeavored to solve the problem: "Why, among men, do those of medium psychic development possess animals of more intelligence than do those of acknowledged culture?" The Arabs possess the most docile animals known, and looking at these animals one may contemplate the value of kindness. The transportation of the Arabian horse from his native soil to this country is attended by a complete change of environment. Coldness or brutality, however, cannot banish entirely the spirit which kindness has fostered, but the quietude of content is replaced by timidity, and confidence in his master is destroyed. This change in disposition is a reflection on our boasted civilization. Referring to methods of training adopted by Mr. Gleason, the writer stated that the lessons of the trainer were not attended by more lasting benefit, because the animals, in many cases, were returned to the same environment which had induced the undesirable habits. The conclusion arrived at by the essayist was "that the Arab, with limited psychical development, places the horse on an equal footing with himself, whereas more civilized man considers himself so far above the animal that the latter's opportunity for psychical development is reduced to a minimum."

Mr. C. A. Boutelle read an instructive paper on "Habit." In the newly-born animal we see many complex movements, some of which are termed automatic activities; others are of reflex nature, and some partake of the nature of both. Some are instinctive, others are rapidly acquired, and then performed with little or no attention, constituting habit, a definition of which involves the fundamental principles of matter. Instincts vary in the individual to adapt themselves to the exigencies of the case. Nerve tissue is endowed with an extraordinary degree of plasticity, so that it may be asserted that "the phenomena of habit in the animal are due to the plasticity of the material of which it is composed." The impressions of outer objects fashion for themselves appropriate paths. Nature has so surrounded the brain about that the only impressions that can be made upon it are through the blood or sensory nerve-roots, and it is to the attenuated currents passing through the latter that the cortex is particularly susceptible. The brain may be considered as an organ through which the currents from the sense organs make with extreme facility paths which do not easily disappear. It must be borne in mind that the incessant nutritive renovation tends to corroborate and fix the modifications in the tissue. Habit, then, simplifies our movements and diminishes the conscious attention with which they are performed. An event calls up its own appropriate successor and without any reference to the conscious will until the whole chain of events necessary for the action are accomplished as if fused into a continuous string, and only requiring the first stimulus. A strictly voluntary act is guided by idea, perception and volition; in habitual action mere sensation is a sufficient guide. The moral aspect of habit was briefly outlined, and reference made to the advantage derived from early developing good habits in man and the lower animals.

Mr. Harri Dell read a paper on the "Evolution of Language." There are indications by which we can approximate the length of time a given faculty has existed in the race, and in following these indications we must admit that language has long been made use of by man. It appears early as a means of expression in the child, is universal, and is not readily lost in sickness. When, however, aphasia is seen we find that the latest acquisitions in language are, in conformity to a psychological law, the first to be parted with, and the primitive gesture-language is retained to the last, and these varied manifestations of this disease aid us in tracing the development of the faculty. There was a period in the early history of the race when speech was unknown, or consisted of but a few imitative sounds, the individual depending on gestures as a means of communication. But means increasing mental development required other means of expression than gestures, and articulate sounds were used more and more until the primitive gesture-language became subservient to speech. Primitive speech was in a large measure composed of words of onomatopœic origin. A similar condition is found in the speech of savages of the present day, and, indeed, many of these reduplicative words are retained in our own language. With developing passions and emotions came the birth and rise of poetry as a means of expression. Poetry was used to perpetuate the deeds of war and the chase, by being handed down from one generation to the next, long before the introductiòn of writing. For the use of articulate speech a cerebral centre is indispensable, also vocal and auditory organs. In many of the lower animals the two latter are found more perfectly developed than in man, but the cortical centre is rudimentary or entirely absent, as shown by the experiments of Ferrier. Thus we see in the lower animals gestures predominating over vocalization, which was probably the condition of the primitive language of man.

A brief discussion followed the reading of the papers, after which the President congratulated the members on the marked development of the Society during the present session, the superiority of the papers, from a literary and scientific standpoint, over those of preceding years, and the valuable acquisitions to the Library.

Lines of work for the next session were suggested and the need of original investigation and experiment pointed out.

